# “*People don’t have the answers*”: A qualitative exploration of the experiences of young people with Long COVID

**DOI:** 10.1177/13591045241252463

**Published:** 2024-05-08

**Authors:** Fiona Newlands, Celine Lewis, Anais d’Oelsnitz, Snehal M Pinto Pereira, Terence Stephenson, Trudie Chalder, Anna Coughtrey, Emma Dalrymple, Isobel Heyman, Anthony Harnden, Tamsin Ford, Shamez N Ladhani, Claire Powell, Kelsey McOwat, Rowan Bhopal, Jake Dudley, Paige Kolasinska, Mohammed Z Muhid, Manjula Nugawela, Natalia K Rojas, Angel Shittu, Ruth Simmons, Roz Shafran

**Affiliations:** 1 Population, Policy and Practice Department, UCL Great Ormond Street Institute of Child Health, UK; 2 NHS North Thames Genomic Laboratory Hub, Great Ormond Street Hospital for Children NHS Foundation Trust, UK; 3 Division of Surgery & Interventional Science, Faculty of Medical Sciences, 4919 University College London, UK; 4 Department of Psychological Medicine, Institute of Psychiatry, Psychology and Neuroscience, King’s College London, UK; 5 Nuffield Department of Primary Care Health Sciences, 228157 University of Oxford, UK; 6 Department of Psychiatry, 2152 University of Cambridge, UK; 7 Immunisation Department, UK Health Security Agency, UK; 8 Paediatric Infectious Diseases Research Group, St George’s University of London, UK

**Keywords:** Children and young people, Long COVID, qualitative, experience, post-COVID condition

## Abstract

Young people living with Long COVID are learning to navigate life with a constellation of poorly understood symptoms. Most qualitative studies on experiences living with Long COVID focus on adult populations. This study aimed to understand the experiences of young people living with Long COVID. Qualitative, semi-structured interviews were conducted (*n* = 16); 11 young people (aged 13–19) and five parents were recruited from the Children and Young People with Long COVID (CLoCk) study (*n* = 11) or its patient and public involvement and engagement (PPIE) group (*n* = 5). Thematic analysis generated four themes: (i) Unravelling Long COVID: Exploring Symptom Journeys and Diagnostic Dilemmas; (ii) Identity Disruption and Adjustment; (iii) Long COVID’s Ripple Effect: the impact on Mental Health, Connections, and Education; and (iv) Navigating Long COVID: barriers to support and accessing services. Treatment options were perceived as not widely available or ineffective, emphasising the need for viable and accessible interventions for young people living with Long COVID.

## Plain language summary

Why was the study done? Capturing the broad impact of Long COVID and the experiences of young people and their families living with persisting symptoms will help to identify the unique needs and challenges experienced by this population and help shape effective treatments going forward. What did the researchers do? Researchers conducted interviews with children and young people living with Long COVID. Parents of young people were also invited to participate to gain a comprehensive understanding of the effects of Long COVID and its impact on the wider family. What did the researchers find? Analysis of 11 interviews with young people and 5 with parents revealed four themes central to young people’s experiences of living with Long COVID relating to unknowns and uncertainties, identity shifts, the impact of symptoms and accessing support. What do findings mean? Findings from the study suggest the implications of Long COVID were far-reaching and impairing. Current treatment options were not perceived as widely available or effective, suggesting a need for further research to develop effective interventions for young people living with Long COVID.

## Introduction

Symptoms enduring after severe acute respiratory syndrome coronavirus 2 (SARS-CoV-2) infection have been variably termed as Post COVID-19 Condition, Post COVID-19 Syndrome, Persistent COVID-19, Long-Haul COVID and Long COVID. The World Health Organization defines the condition as occurring in children and young people (hereafter referred to as ‘young people’) with “a history of confirmed or probable SARS-CoV-2 infection when experiencing symptoms lasting at least 2 months which initially occurred within 3 months of acute COVID-19” (WHO, 2023). Current prevalence estimates of Long COVID in young people vary greatly, ranging from 1-70% (Asadi-Pooya et al., 2021; Hahn et al., 2023; Pellegrino et al., 2022), in part reflecting the diagnostic challenges associated with the condition. Systematic reviews and meta-analyses on the prevalence of these enduring symptoms suggest the condition is complex with multisystemic symptoms including fatigue, headaches, brain fog, post exertional malaise and shortness of breath (Behnood et al., 2022, 2023; Jiang et al., 2023; Lopez-Leon et al., 2022). However, the trajectory of the condition is not clear as symptoms fluctuate and may relapse over time (Ziauddeen et al., 2022).

While our understanding of Long COVID is evolving, there are still many unknowns; there is no specific screening tool or biomarker, and many symptoms are common and non-specific (Espín et al., 2023; Komaroff & Lipkin, 2023). As a result, diagnosis is challenging and generally made by excluding other conditions (Srikanth et al., 2023). This combination of varying prevalence estimates, differing symptom profiles and trajectories, difficulties with diagnoses, lack of specific biomarkers and unknown origins has generated uncertainty and concern over the seriousness of the illness and its potential impact. Given such complexities, it is important to understand the experiences of those living with Long COVID.

A meta-synthesis of qualitative studies on adults with Long COVID identified several findings including challenges living with a complex physical health problem, psychosocial problems including self-identity and emotional disturbance, and issues with slow recovery and rehabilitation (Hossain et al., 2023). These studies focused on adults so do not reflect possible impacts on the developmental milestones which young people must navigate while living with a chronic health condition. Qualitative studies of young people have concentrated on experiences of living through the pandemic and lockdown periods (Clarke et al., 2021; Fisher et al., 2021; McKinlay et al., 2022) but few have explored the experiences of living with persisting symptoms. Those focusing on Long COVID indicate a range of mental health impacts (Messiah et al., 2023) and disruptions to education and schooling (MacLean et al., 2023). However, these studies have focused on specific populations, i.e., young people hospitalised following acute SARS-CoV-2 infection, or on a particular domain of a young person’s life (i.e., education).

Capturing the broad impact of Long COVID and the experiences of young people and their families living with persisting symptoms will help to identify the unique needs and challenges experienced by this population and help shape effective treatments going forward. Therefore, the current study aimed to explore qualitatively the experiences of Long COVID from the perspectives of young people with persisting symptoms and their parents.

## Methods

This study follows the Standards for Reporting Qualitative Research (SRQR) (O’Brien et al., 2014). See supplemental materials.

### Participants

Participants (and their parents) were recruited from the Children and Young People with Long COVID (CLoCk) study or were members of the CLoCk Patient and Public Involvement (PPIE) group. CLoCk is the largest national matched cohort study of young people aged 11–17 in England, capturing data from over 15,000 PCR test-positive young people matched with 15,000 PCR test-negative young people (Stephenson et al., 2021). Pilot interviews were conducted with members of the CLoCk PPIE group with self-reported persistent impairing symptoms. Further potential participants were purposively sampled from CLoCk to include young people who met the Delphi research definition of Long COVID at 3-, 6-, and 12-months after their PCR test (Stephenson et al., 2022a) to capture the experiences of those most impacted by enduring symptoms. To meet this definition, young people had to have at least 1 symptom based on a pre-defined list of 21 common symptoms associated with Long COVID such as shortness of breath, cough, fatigue (Stephenson et al., 2022b), and, using data from the EQ-5D-Y (Wille et al., 2010), be experiencing some/a lot of problems with respect to mobility, self-care, doing usual activities or having pain/discomfort or feeling very worried/sad. Although this definition requires a positive PCR test, this criterion was waived for the current study, but participants were required to self-report persistent impairing symptoms. The decision to waive the requirement of a positive test was twofold: firstly, to address the potential constraints associated with limited testing availability throughout the study period; and secondly, to ensure alignment with the WHO definition of Long COVID, which does not stipulate a positive test result as a prerequisite for diagnosis.

In the current study, the proportion of participants with more than one symptom at 3-, 6-, and 12-months was 100%, 90% and 90% respectively. Symptom data were not available for one participant recruited from the PPIE group. In the CLoCk study itself, the proportion of participants with more than one symptom ranged from 31.8%–55.2%. Further details on the profile of CLoCk participants, including the number of young people experiencing one or more symptoms is provide by Pinto Pereira et al.(Pinto Pereira et al., 2023).

Participants who met the modified Delphi-definition of Long COVID at 3-, 6-, and 12-months after a PCR test were stratified by PCR test status (positive/negative), age, sex at birth, ethnicity and geographical region and invitations to participate were sent in batches (approximately 20 at a time) with the aim of obtaining a diverse range of experiences and to account for the potential number of participants interested in participating. At the point recruitment closed, all participants with sufficient severity and who met who met the modified Delphi-definition of Long COVID at 3-, 6-, and 12-months after a PCR test had been invited to participate. All participants were required to provide their paediatrician or GP contact details and give permission for their primary health care provider to be contacted about any medical or other concerns. A comparison of the study participants to the entire target population (i.e., all those people invited) is presented in Table S2 in the supplemental materials.

Parents of young people were also invited to participate to gain a comprehensive understanding of the effects of Long COVID and its impact on the wider family. Parent participation was not a requirement for young people’s inclusion in the study, meaning young people were able to take part in interviews if their parent was not able to, or did not want to participate.

Principles of information power, the concept that the more information a sample holds, the lower number of participants required, guided recruitment and data collection (Malterud et al., 2016). Researcher notes taken during the interviews were summarised to identify emerging themes. Themes were discussed with the lead researchers’ supervisors and the wider research team. Recruitment ceased once it was established a satisfactory amount of high-quality and in-depth data relating to the study aim had been collected, and no additional themes were emerging.

### Procedure and materials

Ethics approval was granted by Yorkshire and The Humber—South Yorkshire Research Ethics Committee (REC reference: 22/YH/0076; IRAS project ID: 312609).

Young people were contacted via email and asked to contact a member of the research team if they were interested in participating. A researcher responded with details about the study, including an information sheet for young people, and an information sheet for their parents. Parents were asked to contact the researcher if they were interested in participating in the research. All participants were given at least 24 h to decide whether to be involved.

All participants were asked to complete an online consent form and demographics questionnaire before the interview. Where the participant was under 16 years old, assent was obtained from the young person and consent was obtained from a parent or guardian. All interviews were conducted by the lead researcher (FN) on Microsoft Teams, Zoom or telephone. Young people had the choice of having a parent or guardian present during the interview. One young person had a parent present during the interview, and one participated in a joint interview with their parent. Interviews lasted from 23 to 56 min, with an average of 35 min and took place between May 2022 and February 2023.

Interviews followed a topic guide developed by FN, informed by discussion with co-authors RS, IH, TC, and TS. The questions included in the topic guide were informed by existing literature and discussions with experts in the field. The topic guide was reviewed by a researcher from the University of Oxford working on a qualitative study exploring the experience of families living with Long COVID. A first draft of the topic guide was shared with the CLoCk PPIE group for feedback and updated accordingly. The topic guide covered: (1) life pre-pandemic; (2) life during the pandemic; (3) Long COVID symptoms and impact; (4) Long COVID and the family; (5) Long COVID support. Topic guides were refined and updated throughout the data collection process to ensure relevance. See Supplemental materials.

Given the potentially emotive nature of conversations and the potential for participants to reveal significant distress, including suicidal thinking/suicidal intent, a risk management plan was developed, and all interviews took place under the supervision of a clinical psychologist.

All participants were given a £25 gift voucher as per INVOLVE guidance (NIHR, 2022) for their participation. Notes were taken during the interview to aid the analysis process and establish when sufficient data were collected. All interviews were audio-recorded and transcribed verbatim with identifiable information removed.

### Data analysis

Thematic analysis was conducted utilising a codebook methodology (Roberts et al., 2019). Analysis was conducted through an iterative process of coding, revision, and consensus discussions. An initial framework was developed by FN using emerging themes derived from researcher interview summary notes. A subset of transcripts (*n* = 3) were ‘coded’ during which descriptive labels were added to the text and emerging codes were added to the codebook. A second researcher (CL) reviewed the coded transcripts, and the codebook was updated to include a definition and examples. Data were then transferred to NVivo 12 Pro to facilitate the coding process. Once the transcripts had been coded, similar codes were clustered to form categories, and from there, categories were grouped into distinct but related themes. FN led analysis meetings with co-authors (CL, AD, RS, IH, TC, TS) to discuss codes and emerging themes. Interview data from parents and young people were analysed as one dataset.

The lead researcher was a PhD student and had no prior direct contact with young people with Long COVID, thereby limiting the impact of her knowledge on the interviews. Furthermore, she endeavoured to set aside personal beliefs and a priori assumptions to prevent misrepresentation or bias in interpreting participant experiences. She remained conscious of any preconceptions before and during the analysis process and regularly shared reflections on emerging findings with the research team. Additionally, a subset of transcripts was co-coded with a team member possessing different characteristics, fostering a diverse perspective in the coding process.

## Results

### Participant characteristics

157 young people were contacted about the study, and 16 expressed an interest (10.2%). Of those, 8 young people consented and took part in an interview, along with 4 of their parents. Five participants (3 young people and 2 parents) from the PPIE group also participated in interviews. In total, we examined data from 11 young people and 6 parents (See Table 1). Young people’s ages ranged from 13–19 years old (mean age 17.1 years old, (SD = 1.8)). Most young people were female (*n* = 7) and of White (*n* = 5) or Asian/Asian British ethnic background (*n* = 4). Demographic information on sex at birth and ethnicity was unavailable for one young person. In the study cohort, the age distribution mirrored that of the target population, with participants averaging 17.1 years (SD = 1.8), while the target population exhibited a similar mean age of 16.9 years (SD = 1.7). The study cohort had a greater representation of males (27%) compared to the target population (21%), and had a higher proportion of individuals from non-white ethnic backgrounds (45% vs 22% in the target population).

**Table 1.** Participant Demographic Information.

Participant code	Age	Sex at birth	Ethnicity	Recruitment source (CLoCk or PPIE)
Young person demographics				
CYP1	17	Male	Black/African/Caribbean/Black British	PPIE
CYP2	13	NR^1^	NR	PPIE
CYP3	19	Male	Asian/Asian British	PPIE
CYP4	17	Male	White	CLoCk
CYP5	18	Female	Asian/Asian British	CLoCk
CYP6	15	Female	White	CLoCk
CYP7	17	Female	White	CLoCk
CYP8	18	Female	White	CLoCk
CYP9	19	Female	White	CLoCk
CYP10	17	Female	Asian/Asian British	CLoCk
CYP11	19	Female	Asian/Asian British	CLoCk
Parent demographics				
P1	NR	NR	Black/African/Caribbean/Black British	PPIE
P2	NR	NR	NR	PPIE
P4	42	Female	White	CLoCk
P5	61	Female	Asian/Asian/British	CLoCk
P6	47	Female	White	CLoCk
P9	NR	NR	NR	CLoCk

^1^NR = not reported.

Parents ages ranged from 42–61 years (mean age 49.7). Two parents identified as White, one as Asian/Asian British, and one as Black/African/Caribbean/Black British. Half of the parental sample was female (*n* = 3). Demographic information was unavailable for two parents and only partly available for one parent.

Our analysis revealed four inter-related themes: (i) Unravelling Long COVID: Exploring Symptom Journeys and Diagnostic Dilemmas’; (ii) Identity Disruption and Adjustment; (iii) Long COVID’s Ripple Effect: the impact on Mental Health, Connections, and Education; and (iv) Navigating Long COVID: barriers to support and accessing services. The narrative description is below, along with exemplar quotes. Parents’ quotes are identified with a P and young people’s quotes with CYP.

### Theme 1: Unravelling Long COVID: Exploring symptom journeys and diagnostic dilemmas

Central to participants’ experience of living with a novel condition was the sense of coping with unknowns and uncertainties related to the unpredictable nature and progression of Long COVID, and effective management of symptoms.

#### Making sense of symptoms: “You’re just sort of coping with something that’s, very new, and people don’t have the answers”

Young people were experiencing various symptoms, including cognitive issues (e.g., issues with recall and “brain fog”), cardiovascular and respiratory symptoms, loss or altered smell/taste, change in appetite, pain, and dermatological symptoms:

“Sometimes I can’t fall asleep because my legs hurt or my head hurts or like my heart beat is like really fast.” (CYP6)

“I couldn’t do the dishwasher without having to take breaks and getting lightheaded. And when I started to walk, I’d, I started not being able to breath properly.” (CYP9)

“I had extreme fatigue, so like walking down stairs and upstairs would just make me tired and I’d just have to sleep for 7 hours… everything took energy.” (CYP11)

Young people and their parents initially struggled to make sense of what caused these symptoms, what was triggering them, what was making them better or worse and what led to further relapses.

“We had a terrible time a few months ago where it went on for weeks and weeks and weeks, and we didn’t know what to do and what to try, and then it sort of disappeared and then it went away and we thought ‘thank God for that’ and then last week it came it started again out of the blue […] nobody knows why.” (P6)

Young people reflected on the uncertainty of longevity of the condition, questioning *“why is it still here? [...] why can’t it just go away?”* (CYP1) and *“what if it gets worse again today?”* (CYP11). There was a shared sense of frustration from young people and parents about “*how much longer are we gonna keep going with all this before something […] changes*” (P6). Young people struggled with the uncertainty of not knowing whether their symptoms could be “*fixed*” (CYP11) and whether effective treatment options were available:

“Knowing that there’s not a cure. That’s probably like one of the hardest things.” (CYP6)

Long COVID was described as an “*unknown quantity*” (P9) and both young people and parents highlighted the necessity for more research to provide more information and greater recognition of the condition.

#### Difficulties diagnosing Long COVID: “And what would the definition of that be?”

A consequence of the limited understanding of Long COVID is difficulty defining and diagnosing the condition. Young people reported their symptoms had been attributed to other conditions, such as vitamin deficiency and asthma. In some instances, symptoms had been attributed to anxiety, which was a source of frustration.

For one young person, the lack of a clear definition led to challenges in disentangling symptoms of Long COVID with difficulties they experienced because of the pandemic:

“I still got kind of like problems with recall. I don’t know if that’s like just the pandemic, or if erm that’s like from covid” (CYP3).

For another young person, the lack of diagnosis meant they had to continue attending school when unwell:

“I was thinking oh what about if it is just anxiety and I’m making it up but then when I actually got the diagnosis I was like, I knew that something like in the back of my head, I always knew that something wasn’t right” (CYP6)

Having a diagnosis legitimised what this young person was experiencing and they reported that it helped them to deal with the uncertainty of living with a novel condition.

### Theme 2: Identity disruption and adjustment

Parents reported identity shifts in their children, and young people discussed a change in how they saw themselves, making clear distinctions between their current and pre-illness selves.

#### Long COVID and sense of self: “She’s not the same child anymore”

Both young people and parents were learning how to navigate life with long term symptoms, which for young people, meant understanding how to relate to the condition and how it fit with their sense of self. Young people talked about life before- and after-Long COVID describing how the condition had changed their own and other’s perceptions of them:

“it’s just hard to like look back at like pictures and videos of like 2019 and before that and then look at like how you are now […] and feeling just so different.” (CYP6)

Similarly, another young person commented *“everyone, kind of, wants me to be my old self again”* (CYP11). In addition to looking back and mourning their previous selves, participants described being fit and active before the pandemic and found it “*sad”* and “*frustrating*” (CYP6) they were no longer able to do things they had done previously.

#### Acceptance: “She wants to be like her friends, and she’s not”

The impact of living with symptoms made participants feel *“not accepted”* (CYP5) by their peers and they were “*always playing catch-up”* (CYP8). Many young people were gradually accepting what they could and could not easily do and understanding the constraints of their symptoms. This was particularly difficult in the context of milestones in their life:

“It was difficult because at that time we were all turning 18 and, you know, when you turn 18 everyone wants to do more. But it was kind of like, “Oh, we can’t do as much because we’ve still got someone who, who is not fully well” […] So you miss out on a lot of, I don’t know, a lot of events.” (CYP11)

There was a shared sense of not wanting to be *“trapped”* (CYP11) or defined by their symptoms both from a personal and parental perspective. However, this was difficult to avoid given how much the symptoms governed young people’s lives; *“it just kind of rules her life really, unfortunately.”* (P6)

### Theme 3: Long COVID’s ripple effect: The impact on mental health, connections, and education

Parents and young people described the far-reaching impact of Long COVID extending to all areas of the young person’s life, including relationships with friends, family, education, mental health and wellbeing.

#### Impact on relationships: “it puts you in a kind of in a sad position because you don’t really know how to tell people that you want to be there, but you don’t know if you can”

Young people shared various examples to illustrate the impact of living with Long COVID on their social lives. For example, feeling isolated and lonely, not wanting to socialise as much, and not enjoying the same social activities as before the pandemic and the onset of Long COVID. Young people had lost contact with friends because they had “*not forgotten but just don’t really know what to say*” (CYP6). Young people reflected on the social opportunities they lost with friends as they could not attend school.

Both young people and parents reported Long COVID was also having an impact on family life. For example, one young person felt their relationship with their parent had been negatively affected by the parent’s focus on their symptoms, which resulted in overwhelming worry. Additionally, a parent noted how the impact of their child’s symptoms meant they were restricted in what they were able to do as a family, which was difficult for the whole family unit.

#### Impact of Long COVID on mental health: “It’s a physical thing, but […] the mental impact’s gonna linger, I think, a lot longer”

A further impact of Long COVID was experiencing increased feelings of anxiety. One source of this anxiety was being unable to do ‘normal’ activities anymore. One young person who felt their Long COVID symptoms had improved was still unable to exercise because they were anxious they might become out of breath and a parent whose daughter continued to experience nosebleeds after COVID-19 felt she had become anxious about socialising with friends.

Another source of anxiety for young people experiencing Long COVID symptoms was their general health:

“those feelings of just feeling generally unwell, they quite often send me into bit of a spiel like anxiety, or health anxiety type thing […] obviously feeling tired is a symptom of a lot of things, so I’d go on a mad google search and you know and diagnose myself with like sepsis and stuff.” (CYP8).

Experiencing a common and non-specific symptom was leading to heightened concerns about their health more generally, prompting feelings of anxiety.

#### Impact on education: “Am I gonna forget really crucial details just because I’ve got this fog?”

Anxiety around education and exams was a shared concern amongst young people and parents. The main concerns were the impact of brain fog and problems with recall on exams:

“I think definitely the recall’s made me more anxious, and especially with like my final exams at school coming […] poor recall when you’re kind of doing exams doesn’t make it easy at all.” (CYP3)

Young people discussed how difficulties with concentration and tiredness impacted their ability to engage in school. Young people had missed days, weeks or months off school, with one young person reporting an impact on grades. When participants were well enough to go to school, they had to miss lessons because of GP or hospital appointments. For one family, the result of missing so much school was being referred to the local authority who threatened to take the parents to court.

### Theme 4: Navigating Long COVID: barriers to support and accessing services

Despite young people and parents reporting Long COVID’s impact, many struggled to access formal support to help manage symptoms.

#### Access to support: “There’s just no kind of guidance or legislation in place for children with an illness of this kind of nature”

Some services, including schools, had been unable to adapt to provide the necessary support for young people experiencing Long COVID symptoms. Parents described challenges “*battling for recovery support*” (P6) and were grateful for those healthcare professionals who advocated and “*pushed forward for Long COVID testing*” (P9). For those services that could adapt, other barriers impacted the effective practice or provision of support, including paying for private treatments or long waitlists. Some young people who were able to receive treatment faced difficulties due to the nature of their symptoms. For example, one parent shared their child was too tired to commit to attending regular counselling sessions which meant they had to stop attending.

#### Treatment and management: “Adapt […] we adapt”

Looking to the future, young people and parents addressed their attitudes towards treating and managing Long COVID. Both groups shared examples of the self-management treatments they’d used to address symptoms including techniques they believed would and boost their immune systems, taking painkillers, using ice packs, cold flannels and head massages.

The importance of education was identified by some young people, who felt it was important to have information about common Long COVID symptoms. Young people felt the more information they had about Long COVID and its associated symptoms, the more reassured they would feel:

“knowing there are other people are having the same symptoms and that it’s not something kind of more sinister [...] So, you know maybe like a clear symptom maybe, that kind of things normal that would be reassuring” (CYP8).

For one family, peer support via an online advocacy group was integral to coping with Long COVID. Peer support was described as a “*lifeline*” and “*invaluable*”, offering a “*safe space*” (P6) to share the types of treatments tried, progress made and mutual support.

## Discussion

This is one of the first qualitative studies to look at the impact and experiences of young people affected by Long COVID. Analysis of 11 interviews with young people and 5 with parents revealed four themes central to young people’s experiences of living with Long COVID relating to unknowns and uncertainties, identity shifts, the impact of symptoms and accessing support. When discussing their experience of Long COVID, young people and parents shared many uncertainties relating to symptom profile, trajectory, and prognosis. This uncertainty was compounded by the lack of evidence from research and a lack of health professionals’ guidance on managing symptoms effectively. Similar concerns have been cited in studies of adults with Long COVID who report feelings of uncertainty due to variations in patterns of health problems, unknowns of recovery, and relapse of symptoms (Hossain et al., 2023). ‘Illness uncertainty’ is a concept that has been explored in the wider literature on long-term physical health problems, with theories highlighting the emotional aspects of uncertainty, including anxiety, stress and confusion that accompany the unpredictable nature of illness (Mishel, 1990). Such theories highlight the need to not only address information gaps relating to the illness but also acknowledge the psychological challenges individuals face when navigating their symptoms.

Studies on long-term physical health conditions report a link between an individual’s ability to tolerate uncertainty and their psychological wellbeing; those who are less able to tolerate uncertainty report poorer outcomes and quality of life (Gibson et al., 2023). The potential impact on emotional wellbeing and coping is particularly worrying when considered in the context of Long COVID, where young people are left to navigate life with a poorly understood condition with many unknowns related to the cause and longevity of symptoms. Freeston and Komes (2023) suggest individuals with high uncertainty intolerance try to “maximise personal safety” and withdraw from activities to preserve their wellbeing. This reflects the experiences of young people in the current study who no longer engaged in ‘normal’ activities over fear symptoms would relapse or worsen. Participants were learning to live with this uncertainty and adapt to their new ‘limits’; they were caught between not wanting to be defined by their symptoms and acknowledging that they were different post-illness.

Young people described a change in their identity, feeling markedly different from their peers and struggling with a sense of disconnection and non-acceptance. This change in identity was reflected in their narratives, where they articulated feelings of detachment from friends and a sense of being alienated from others. Part of this altered identity involved missing out on typical adolescent activities, including attending school and concern over a potential decline in academic performance. For young people in an important transitional point of life, educational setbacks are particularly challenging, causing apprehension about the potential long-term consequences.

Given the impact of symptoms on young people, participants were keen to have access to treatment and services, but perceptions of services’ ability to adapt to meet the needs of young people and provide effective support was mixed. Participant narratives highlighted the length of time it took from symptoms first being acknowledged to when a final diagnosis was eventually made, possibly reflecting the novel nature of the condition and a diagnosis generally being made on exclusion. Amidst these challenges, several participants emphasised the pivotal role of healthcare professionals who proactively advocated for their patients’ needs and facilitated access to essential resources. Parents expressed gratitude towards healthcare professionals who actively pursued Long COVID testing for their children, highlighting the critical importance of advocacy in ensuring timely diagnosis and appropriate management.

Current assessment and management approaches for Long COVID in young people emphasises supported self-management and multidisciplinary rehabilitation support including physical and cognitive assessments, alongside diagnostic tests and management strategies (Johnston et al., 2024; Wacks et al., 2024). However, the absence of available research and evidence about effective treatment options meant young people were finding what worked to alleviate symptoms as they went. As described in the adult literature (Hossain et al., 2023; Russell et al., 2022), young people and their parents used self-management techniques, shared ideas among patient groups and found validation and support from online communities.

### Strengths and limitations

This is one of the first studies to investigate the experience of young people living with Long COVID, capturing the broad impact of the condition on different areas of their lives. Topic guides were informed by the CLoCk PPIE group to ensure questions were relevant to young people living with Long COVID. Participants were also recruited from the wider CLoCk study (Stephenson et al., 2021), which initial analysis indicates is broadly representative of the population of young people in England (Rojas et al., 2023).

However, a relatively high proportion of young people did not respond to the invitation to be involved in the study. The non-responders group may have included young people who were too unwell to participate in an interview, so the study may not have captured the experiences of those with very severe Long COVID. As with many other research studies, most participants were female and identified as White British and, therefore, may not be representative of younger children and those from different ethnic backgrounds.

## Conclusion

The participants in this study described having to navigate the unpredictable nature of multiple and changing symptoms, of varying severity. Findings from the study suggest the implications of Long COVID were far-reaching and impairing. The many uncertainties surrounding this poorly understood condition intensified the impact on young people’s wellbeing. Current treatment options were not perceived as widely available or effective, suggesting a need for further research to develop effective interventions for young people living with Long COVID.

## Supplemental Material

Supplemental Material - “*People don’t have the answers*”: A qualitative exploration of the experiences of young people with Long COVID Supplemental Material for “*People don’t have the answers*”: A qualitative exploration of the experiences of young people with Long COVID by Fiona Newlands, Celine Lewis, Anais d’Oelsnitz, Snehal M Pinto Pereira, Terence Stephenson, Trudie Chalder, Anna Coughtrey, Emma Dalrymple, Isobel Heyman, Anthony Harnden, Tamsin Ford, Shamez N Ladhani, Claire Powell, Kelsey McOwat, Rowan Bhopal, Jake Dudley, Paige Kolasinska, Mohammed Z Muhid, Manjula Nugawela, Natalia K Rojas, Angel Shittu, Ruth Simmons and Roz Shafran in Clinical Child Psychology and Psychiatry
