# Supplementary material for: “People don’t have the answers”: A qualitative exploration of the experiences of young people with Long COVID
Source: Clin Child Psychol Psychiatry. 2024 May 8;29(3):783–98. doi: 10.1177/13591045241252463 (PMC11188547; doi:10.1177/13591045241252463)
Supplement: Supplemental Material - “People don’t have the answers”: A qualitative exploration of the experiences of young people with Long COVID [file sj-pdf-1-ccp-10.1177_13591045241252463.pdf]

"*People don't have the answers*": A qualitative exploration of the experiences of young people with Long COVID. Newlands et al.

Table 1: Standards for reporting qualitative research (SRQR) checklist

No.	Topic	Items	Page no.
**Title and Abstract**			
S1	Title	Concise description of the nature and topic of the study Identifying the study as qualitative or indicating the approach (e.g., ethnography, grounded theory) or data collection methods (e.g., interview, focus group) is recommended	Page 1
S2	Abstract	Summary of key elements of the study using the abstract format of the intended publication; typically includes background, purpose, methods, results, and conclusions	Page 5
**Introduction**			
S3	Problem formulation	Description and significance of the problem/phenomenon studied; review of relevant theory and empirical work; problem statement	Page 6&7
S4	Purpose or research question	Purpose of the study and specific objectives or questions	Page 7
**Methods**			
S5	Qualitative approach and research paradigm	Qualitative approach (e.g., ethnography, grounded theory, case study, phenomenology, narrative research) and guiding theory if appropriate; identifying the research paradigm (e.g., postpositivist, constructivist/ interpretivist) is also recommended; rationale	Page 8-11
S6	Researcher characteristics and reflexivity	Researchers' characteristics that may influence the research, including personal attributes, qualifications/experience, relationship with participants, assumptions, and/or presuppositions; potential or actual interaction between researchers' characteristics and the research questions, approach, methods, results, and/or transferability	Page 8-11
S7	Context	Setting/site and salient contextual factors; rationale	Page 8-11
S8	Sampling strategy	How and why research participants, documents, or events were selected; criteria for deciding when no further sampling was necessary (e.g., sampling saturation); rationale	Page 8
S9	Ethical issues pertaining to human subjects	Documentation of approval by an appropriate ethics review board and participant consent, or explanation for lack thereof; other confidentiality and data security issues	Page 9
S10	Data collection method	Types of data collected; details of data collection procedures including (as appropriate) start and stop dates of data collection and analysis, iterative	Page 9-11

		process, triangulation of sources/methods, and modification of procedures in response to evolving study findings; rationale	
S11	Data collection instruments and technologies	Description of instruments (e.g., interview guides, questionnaires) and devices (e.g., audio recorders) used for data collection; if/how the instrument(s) changed over the course of the study	Page 10
S12	Units of study	Number and relevant characteristics of participants, documents, or events included in the study; level of participation (could be reported in results)	Page 11-12
S13	Data processing	Methods for processing data prior to and during analysis, including transcription, data entry, data management and security, verification of data integrity, data coding, and anonymization/de-identification of excerpts	Page 11
S14	Data analysis	Process by which inferences, themes, etc., were identified and developed, including the researchers involved in data analysis; usually references a specific paradigm or approach; rationale	Page 11
S15	Techniques to enhance trustworthiness	Techniques to enhance trustworthiness and credibility of data analysis (e.g., member checking, audit trail, triangulation); rationale	Page 11
**Results/findings**			
S16	Synthesis and interpretation	Main findings (e.g., interpretations, inferences, and themes); might include development of a theory or model, or integration with prior research or theory	Page 9 – 16
S17	Links to empirical data	Evidence (e.g., quotes, field notes, text excerpts, photographs) to substantiate analytic findings	Page 10 – 16
**Discussion**			
S18	Integration with prior work, implications, transferability, and contribution(s) to the field	Short summary of main findings; explanation of how findings and conclusions connect to, support, elaborate on, or challenge conclusions of earlier scholarship; discussion of scope of application/generalizability; identification of unique contribution(s) to scholarship in a discipline or field	Page 11-20
S19	Limitations	Trustworthiness and limitations of findings	Page 23
**Others**			
S20	Conflict of interest	Potential sources of influence or perceived influence on study conduct and conclusions; how these were managed	Page 4
S21	Funding	Sources of funding and other support; role of funders in data collection, interpretation, and reporting	Page 2-3

Topic guide

Stem	Follow-up questions and prompts
**Section 1: Pre pandemic**	
Can you tell me about you day-to day life before the COVID-19 pandemic?	• *School*<br>• *Friends*<br>• *Family*<br>• *Hobbies*
How would you describe your general health & wellbeing before the pandemic?	*Can you tell me about any health or wellbeing concerns you had prior to the pandemic?*<br><br>*Can you tell me a bit more about that?*<br>• *Specific problems*<br>• *What impact were they having?*<br>• *How long were you experiencing them?*<br>• *Sought help for problems?*
**Section 2: During the pandemic**	
Can you tell me about you day-to day life during the COVID-19 pandemic?	• *School*<br>• *Friends*<br>• *Family*<br>• *Hobbies*
How would you describe your general health and wellbeing **during** the pandemic?	*Can you tell me about any health or wellbeing concerns you had during the pandemic?*<br><br>*Can you tell me a bit more about that?*<br>• *Specific problems*<br>• *What impact were they having?*<br>• *How long were you experiencing them?*<br>• *Sought help for problems?*
**Section 3: long COVID**	
Can you tell me about when you first contracted COVID-19? How did you first notice or become aware that you might have contracted the virus?	• *How many times do you think you've contracted the virus?*<br>• *Were tests available at the time?*<br>• *Did you think you contracted COVID even though you didn't test positive?*
Can you describe the initial onset of your Long COVID symptoms? How did they manifest, and how severe were they?	• *What symptoms of Long COVID did you have/ do you have currently?*<br>• *Have you recovered from Long*

	COVID?<br>• *How do you know you have recovered?*
Can you describe the symptoms you are currently experiencing due to Long COVID?	• *How have these symptoms changed over time?*<br>• *How severe are your symptoms when they are at their worst?*<br>• *Is there anything that made them better/worse?*<br>•
Could you describe the ways in which Long COVID has influenced or affected different aspects of your life? How have these effects evolved or changed since you first experienced Long COVID symptoms?	• *Mental health and wellbeing*<br>• *Friendships*<br>• *School*<br>• *Hobbies*<br>• *Family relationships*<br>• *Eating (normally and enjoyment)*<br>• *Sleep*<br>• *Other*
**Section 4: Long COVID and your family**	
Has anyone else in your family experienced persisting symptoms after contracting COVID-19?	• *Could you describe how this has influenced your family?*
**Section 5: Long COVID support**	
Have you been to see anyone about your long COVID symptoms? e.g., your GP, been to hospital, a long COVID hub, CAMHS, Occupational therapist, Reflexologist, Osteopath, school counsellor	• *Can you tell me a bit more about that? How was your experience?*<br>• *Have you noticed any changes or improvements in your symptoms after undergoing these treatments?*<br>• *If no, why have you not been to see a health professional?*
What kind of information or resources do you feel would have been helpful to you when you were first diagnosed or experiencing Long COVID symptoms?	• *How do you think these resources could have helped you/ your symptoms?*
Are you a member of any Long COVID support/ advocacy groups?	• *Can you tell me about the support you receive from the group?*<br>• *How has being a member of these groups impacted you?*

Table 2: Comparison of study participants compared to the target population (i.e., those invited)

		Study participants (n=11)*	Target population (n=157)
**Age Mean (SD)**		17.1 (1.8)	16.9 (1.7)
**Sex at birth**	Female	7 (64%)	124 (79%)
	Male	3 (27%)	33 (21%)
	Unknown	1 (9%)	0 (0%)
**Ethnicity**	Asian/ Asian British	4 (36%)	20 (13%)
	Black/ African/ Caribbean/ Black British	1 (9%)	5 (3%)
	Mixed	0 (%)	9 (6%)
	White	5 (45%)	121 (77%)
	Unknown	1 (9%)	0 (0%)

*demographic information was not available for one of the study participants
